# Respiratory Motion Compensation Using Diaphragm Tracking for Cone-Beam C-Arm CT: A Simulation and a Phantom Study

**DOI:** 10.1155/2013/520540

**Published:** 2013-06-06

**Authors:** Marco Bögel, Hannes G. Hofmann, Joachim Hornegger, Rebecca Fahrig, Stefan Britzen, Andreas Maier

**Affiliations:** ^1^Pattern Recognition Lab, Friedrich-Alexander-University Erlangen-Nuremberg, 91058 Erlangen, Germany; ^2^Department of Radiology, Lucas MRS Center, Stanford University, Palo Alto, CA 94304, USA; ^3^Siemens AG, Healthcare Sector, 91301 Forchheim, Germany

## Abstract

Long acquisition times lead to image artifacts in thoracic C-arm CT. Motion blur caused by respiratory motion leads to decreased image quality in many clinical applications. We introduce an image-based method to estimate and compensate respiratory motion in C-arm CT based on diaphragm motion. In order to estimate respiratory motion, we track the contour of the diaphragm in the projection image sequence. Using a motion corrected triangulation approach on the diaphragm vertex, we are able to estimate a motion signal. The estimated motion signal is used to compensate for respiratory motion in the target region, for example, heart or lungs. First, we evaluated our approach in a simulation study using XCAT. As ground truth data was available, a quantitative evaluation was performed. We observed an improvement of about 14% using the structural similarity index. In a real phantom study, using the artiCHEST phantom, we investigated the visibility of bronchial tubes in a porcine lung. Compared to an uncompensated scan, the visibility of bronchial structures is improved drastically. Preliminary results indicate that this kind of motion compensation can deliver a first step in reconstruction image quality improvement. Compared to ground truth data, image quality is still considerably reduced.

## 1. Introduction

C-arm CT has enabled reconstruction of 3D images during medical procedures, for example, cardiac interventions. However, the rather long acquisition time of several seconds may lead to motion artifacts, such as motion blur and streaks. These artifacts are very problematic in many clinical applications. The commonly used technique to reduce the influence of respiratory motion during cardiac procedures is the so-called single breath-hold scan. This approach requires the patient to hold his breath for the duration of the scan. Unfortunately, this technique does not guarantee perfect results. Jahnke et al. have measured residual respiratory motion in almost half of their test group containing 210 people [1]. We have two main applications in the focus of our work. One is the improvement of cardiac C-arm CT. While compensation of the motion of the heart has been investigated intensively in the literature [2–4], the problem of respiratory motion during cardiac C-arm CT is much less frequently addressed. Residual respiratory motion during the cardiac scan causes a considerable reduction in image quality.

Motion artifacts are also very problematic in pulmonary procedures. In order to analyze the malignancy of a pulmonary tumor, a sample has to be extracted. A bronchoscope is inserted through the patient's nose and has to be navigated through the bronchial tree towards the tumor. This procedure requires an accurate plan of the bronchial tree. However, most tumors are only accessible through bronchi with diameters of less than 2 mm. Therefore, we require accurate imaging without motion blur, otherwise the small bronchi are not visible. Respiratory motion can be reduced with a jet ventilator that inflates the lung with oxygen. There are two downsides to this approach. The efficiency of this approach depends on the amount of pressure that is used. While too small pressure results in residual motion, too high pressure may cause rupture and pneumothorax in consequence. Additionally, natural reflexes of the human body may also cause residual motion. Therefore, it is necessary to develop new methods to estimate and compensate respiratory motion in C-arm CT. There are many different ways to acquire respiratory signals. Most are based on additional equipment, for example, Time-of-Flight or stereo vision cameras [5]. Other techniques try to extract the respiratory signal directly from the projection images. Using an image-based approach the extracted respiration signal is perfectly synchronized with the projection images. Image-based respiratory motion extraction often relies on tracking of fiducial markers in the projection images [6, 7]. Wang et al. have shown that the motion of the diaphragm is highly correlated to respiration-induced motion of the heart [8]. Sonke et al. propose to extract a 1D respiration signal by projecting diaphragm-like features on the superior-inferior axis and selecting the features with the highest temporal change [9]. However, the downside of this approach is that the extracted signal is not the real respiration signal. Due to perspective projection, the projected amplitude depends on the C-arm rotation angle. Kavanagh et al. recently proposed a similar approach analyzing the intensity values between projections that works without any external or internal oscillating structures [10]. Another recent approach by Chen and Siochi tracks the diaphragm using a combination of Hough Transform and Active Contours and an interpolated ray-tracing algorithm to estimate a respiration signal [11]. Vergalasova et al. proposed a Fourier Transform based approach that also works without any markers [12]. 

In this paper, we propose to estimate respiratory motion by tracking the diaphragm in the projection image sequence. The tracked position of the diaphragm top is used to compute a 1D respiration signal, which is then incorporated into the reconstruction algorithm to compensate for respiratory motion in the volume of interest.

## 2. Materials and Methods

 The proposed method is composed of three major steps that are each discussed in the following sections. In the first step, the contour of the diaphragm is tracked throughout the entire projection image sequence. Based on this tracking, we are able to obtain the 2D projection of the diaphragm top for each image. In the second step, a motion corrected triangulation approach is used to compute the 3D position of the diaphragm top for each projection. Assuming superior-inferior respiratory motion, the 1D respiration signal is extracted. In the final step, the respiration signal is used to compensate for respiratory motion during reconstruction.

### 2.1. Diaphragm Tracking

 We introduced a model-based tracking method that is able to accurately track the contour of a user-selected hemidiaphragm in a set of rotational projection images [13]. Compared to other tracking-based methods, for example, fiducial markers, the shape we want to track is not unique. The diaphragm appears as two similar shaped hemidiaphragms. Therefore, it is necessary for the user to select the one to be tracked. The user selects a point roughly located at the top of the desired contour. Subsequently, we define a rectangular Region of Interest (ROI) symmetrically around the selection. We propose an ROI of size 250 × 55 for projection images of size 640 × 480. The image is then preprocessed using a gaussian low-pass filter and the Canny edge detector.

In the next step, the Random Sample Consensus (RANSAC) [14] is used to fit a parabolic curve to the obtained set of edge points. RANSAC can deal with datasets with large percentages of gross errors and is thus the ideal choice to fit a model to our very noisy set of points. The aim of this method is to model the diaphragm as a quadratic function *v* = *au* ^2^ + *bu* + *c*, where *u* and *v* are the detector coordinates. The parabolic model is a good fit for the top of the diaphragm, in which we are interested in. The asymmetry in lower parts of the diaphragm does not affect our model, as our ROI limits the estimation to the top region. The parabolic model allows for very fast model estimation, as well as a simple extraction of the diaphragm vertex. RANSAC has to estimate the three parameters *a*, *b*, and *c*. In the first step, three random points are selected. The model estimation is then formulated as the following optimization problem:

(1) $$\begin{array}{c} \overset{3}{\underset{i=1}{\sum }}{\left(a\cdot u_{i}^{2}+b\cdot u_{i}+c-v_{i}\right)}^{2}\to \min. \end{array}$$

A total of *N* models are estimated, each based on different randomly selected points, and evaluated to determine the best one. A model's quality is defined by the number of inliers. An inlier is a point that lies within a predefined distance to the model. Since an accurate model is desired, we only consider points with a one pixel distance to the model inliers. Subsequently, out of the *N* estimated models we choose the one with the highest number of inliers. Assuming small motion between subsequent frames, the contour is tracked by calculating the current contour's vertex and using it as the start point in the subsequent frame.

One additional important optimization is made. Instead of continuing to use the rectangular ROI, we restrict it to a parabolic ROI based on the model from the previous frame. The parabolic region should be of sufficient height to contain the current diaphragm. In our experiments we used a parabolic region of 21 pixels height, centered around the previous model. This approach decreases the number of points we have to consider in the model estimation drastically.

To guarantee accurate tracking in projections where both hemidiaphragms are visible in the ROI, we propose additional constraints based on the small motion assumption and prior knowledge: (i) the horizontal motion of the contour is limited by the average motion in previous frames, (ii) deformation of the contour (defined by the change of model parameter *a*) is limited to 5% compared to the previous model, and (iii) the direction of horizontal motion can be derived from patient position and C-arm rotation. Suppose acquisition starts from the right lateral view. Rotating towards the frontal view, the contour of the right hemidiaphragm will move to the left, whereas the contour of the left hemidiaphragm moves to the right. From the frontal position to the left lateral position this motion is reversed. We can now enforce the model to move in one direction until the turning point and then move in the opposite direction. However, since the diaphragm is deforming during respiration, it is better to loosen this constraint, by allowing free motion around the turning point. Allowing free motion in the frontal views is not problematic as the hemidiaphragms do not overlap or interfere in these views.

### 2.2. Triangulation and Signal Extraction

 The result of the diaphragm tracking is a parabolic model of the hemidiaphragm for each image. Our approach relies on the assumption that the projection of the 3D diaphragm top coincides with the top of the 2D diaphragm contour. However, this assumption is quite restrictive. Based on this assumption, we are able to reconstruct the 3D position using multiview triangulation. We use triangulation to acquire a motion signal in millimeter that we can use for motion compensated reconstruction. However, triangulation algorithms are designed for static scenes and yield inaccurate results when used for dynamic scenes. For triangulation of dynamic scenes we propose the following four step process:

1. select a pair of images,
2. rectification of the image planes [15],
3. motion correction,
4. triangulation [16].

First, we select two images with the contour vertices $\tilde{g}={\left({\tilde{g}}_{u},{\tilde{g}}_{v},1\right)}^{T}$ and ${\tilde{g}}^{\prime }={\left({\tilde{g}}_{u}^{\prime },{\tilde{g}}_{v}^{\prime },1\right)}^{T}$. Ideally, the selected images should be acquired from orthogonal views, as using nonorthogonal images results in lower triangulation accuracy. The second step is essential for the subsequent motion correction. The rectification algorithm by Fusiello et al. projects the two projection images onto a common image plane, so that their epipolar lines become parallel and horizontal [15]. Subsequently, the transformed images have one very important feature: assuming no motion, the projections of a specific point have the same vertical coordinate in both image planes. Thus, after transforming the detected point correspondences, any residual difference in their vertical coordinates must be caused by respiratory motion during image acquisition. Therefore, we can eliminate the respiratory motion of this image pair in the third step. We choose the first point $\tilde{g}$ as the reference and the corresponding point in the second image is set to

(2) $$\begin{array}{c} {\tilde{g}}^{\prime }={\left({\tilde{g}}_{u}^{\prime },{\tilde{g}}_{v},1\right)}^{T}. \end{array}$$

Finally, we use the transformed and motion corrected point correspondences to triangulate the corresponding 3D point. In this work a simple iterative Linear-Eigen approach, as proposed by Hartley and Sturm [16], has yielded excellent results.

After we triangulate a 3D point corresponding to each image, we can now compute the respiration signal. Since respiratory motion is generally considered as a mainly translational motion along the superior-inferior axis, we compute the 1D respiration signal $\hat{r}$ as

(3) $$\begin{array}{c} {\hat{r}}_{i}=z_{\text{ref}}-z_{i}, \end{array}$$

with *z*_ref_ as the *z*-coordinate of the reference point and *z*_*i*_ as the *z*-coordinate of the triangulated point corresponding to a projection image *i*. Finally, the resulting signal is smoothed using a gaussian low-pass filter.

### 2.3. Motion Compensated Reconstruction

The signal is now included in the reconstruction process. Schäfer et al. recently introduced a motion compensated backprojection algorithm for cone-beam CT [17]. As we only utilize a rigid 1D motion vector field, we use a simplified version of their approach.  Algorithm 1 shows the motion compensated pixel-driven reconstruction algorithm. For each projection, each voxel is projected on the detector to get the update value. Instead of regularly updating the volume, we first compensate for respiratory motion by shifting the voxel back to its reference position using the estimated signal. Then, we update the corrected voxel. Therefore, we are able to obtain a reconstruction at the reference time we selected for the respiration signal. So far, the proposed method assumes a constant shift when compensating the respiratory motion of the heart. 

## 3. Results and Discussion

 The proposed methods were evaluated on the simulated XCAT software phantom [18, 19] and the artiCHEST phantom (PROdesign GmbH, Germany), a porcine lung phantom that allows simulation of respiratory motion. Note that a fraction of the experimental results were already published in two short conference abstracts [13, 20].

### 3.1. XCAT Software Phantom

 The first evaluation of this work was carried out on the XCAT software phantom [18, 19]. The purpose of this evaluation is to compensate respiratory motion of the heart. The XCAT phantom was created with respiratory motion only. We simulated an acquisition time of four seconds with one full respiration cycle. The simulated motion model was rigid; both heart and diaphragm moved about 2.3 cm along the superior-inferior axis; the rest of the scene was static. A detector of size 640 × 480 px was simulated with a resolution of 0.616 mm/px. 200 projections were acquired with an average angular increment of 1.0°. As ground truth, we used the reconstruction of an XCAT dataset that was simulated without respiratory motion.

**Figure 1:**
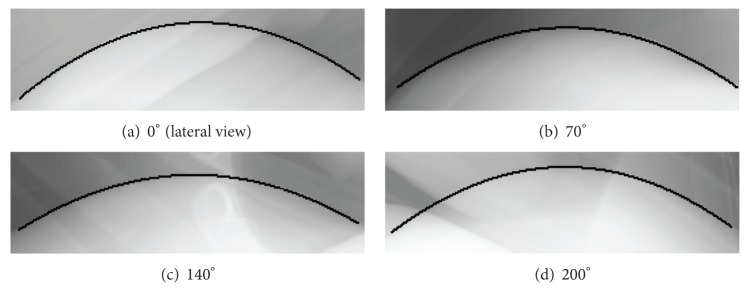
Diaphragm tracking on simulated XCAT data. Images (a)–(d) show projection images acquired from different angles.

The diaphragm tracking method was evaluated on the left and right hemidiaphragms in XCAT projection data [13]. We were able to track the vertex of the diaphragm contour with sub-pixel accuracy. We observed a Euclidean distance of the right vertex to the correct vertex of 0.45 ± 0.56 pixels and 0.75 ± 0.84 pixels for the left vertex, respectively [13].  Figure 1 shows the estimated parabolic model of the diaphragm from four different views of the XCAT phantom.

**Figure 2:**
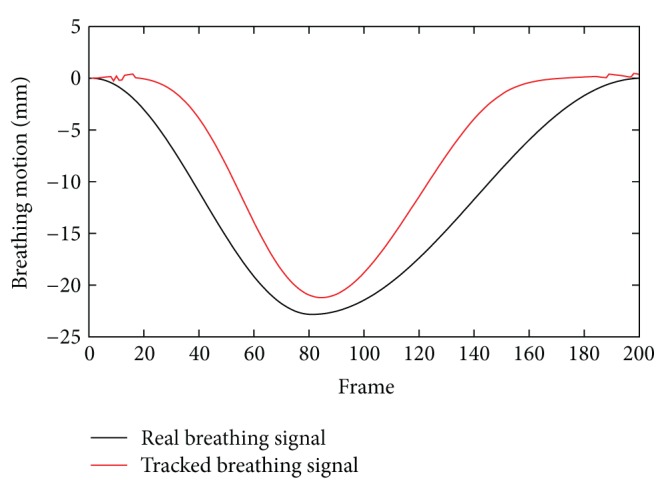
Comparison of the extracted diaphragm motion signal and the actual respiration signal of the simulated XCAT phantom. The amplitude of the signal cannot be estimated accurately, as the projections of the diaphragm top do not coincide with the 2D contour.

Figure 2 shows the extracted signal based on triangulation of the diaphragm tracking results. As previously noted, our approach depends on the assumption that the projection of the diaphragm top lies on the 2D contour. However, this is a strong assumption that is not always fulfilled. In fact, the correct projection of the diaphragm top is often located below the contour, due to perspective projection. This results in inaccuracies in the estimated amplitude of the signal, caused by triangulation with false point correspondences. As shown earlier, we have a deterministic error in the reconstruction of the height of the diaphragm top caused by the perspective projection in the cone-beam data. However, this is only a limitation of the current triangulation approach and not a limitation of the method in general. Thus, we expect to get results that are similar to the correct correspondence case once we have solved this problem. In order to assess the accuracy of the triangulation approaches without the effect of false point correspondences, we tested the methods using the projections of the diaphragm top of the same XCAT simulation with respiratory motion as input for the rectified triangulation algorithm. Therefore, we can test the performance of our algorithm assuming a correct tracking. We triangulated the 3D position of the diaphragm top for each projection image using a second image with a certain angular offset (10°–90°). The triangulated 3D point was compared to the actual 3D position of the diaphragm top at the time the projection was acquired. As results in Table 1 show, our rectified iterative approach provides submillimeter accuracy even for image pairs with low angular offset, whereas the average error of the standard triangulation approach without rectification and motion correction is about 10% of the total respiratory motion. These results indicate that we are able to reconstruct an accurate respiration signal, given the correct positions of the diaphragm top in the 2D projection images. Our methods were tested on an Intel Xeon X5450 CPU. Even though it is not yet optimized and only implemented in Java, our proposed method is very efficient, yielding a computation time of the diaphragm tracking combined with triangulation of approximately 10 seconds, using 20000 RANSAC iterations per frame. This corresponds to a runtime of approximately 50 ms per frame. 

**Figure 3:**
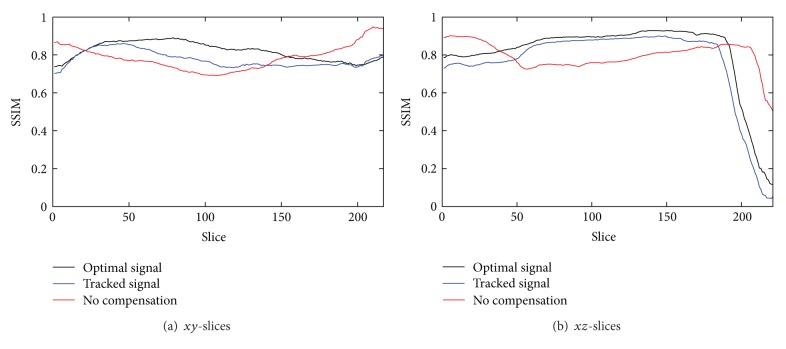
Structural similarity index of the heart volume for *xy*- and *xz*-slices. The uncompensated reconstruction shows better results in the beginning and the end, as the heart is only of small size in these slices.

**Figure 4:**
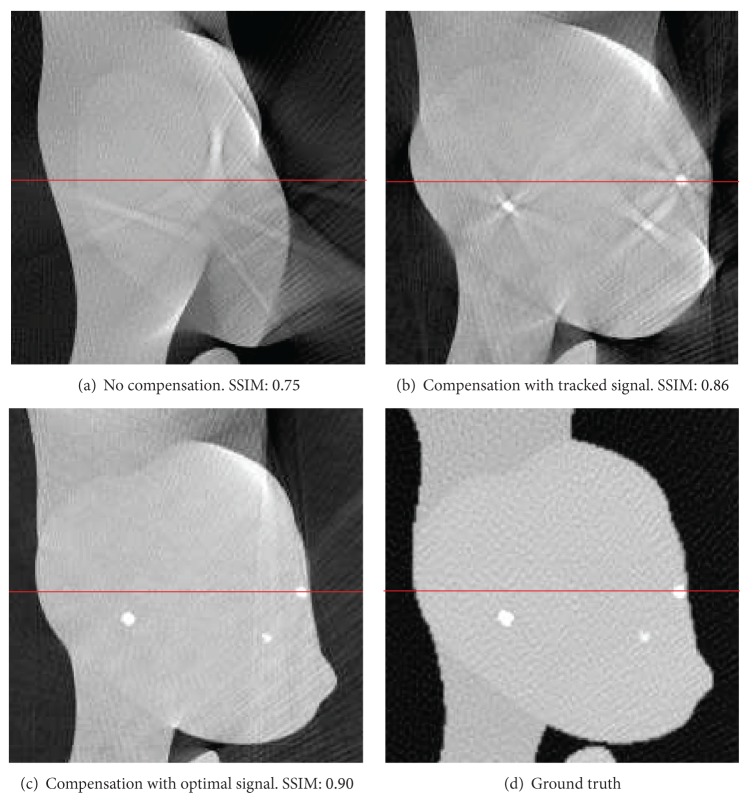
Comparison of *xy*-slice 70 of compensated and uncompensated volumes (cf.  Figure 3(a)). Simulated high-contrast heart lesions further illustrate the improved image quality. Line profiles were taken at the position of the red lines.

**Figure 5:**
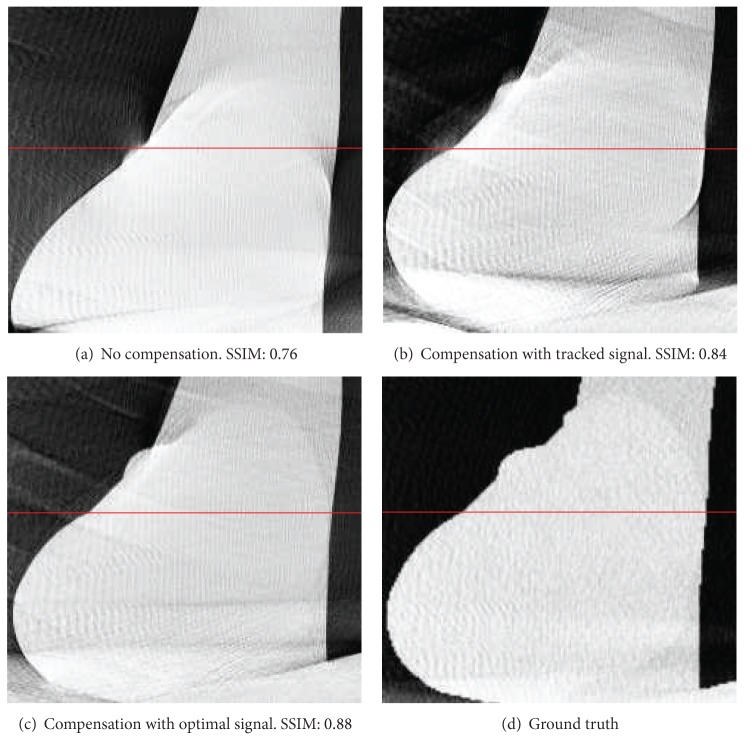
Comparison of *xz*-slice 60 of compensated and uncompensated volumes (cf.  Figure 3(b)). Line profiles were taken at the position of the red lines.

**Figure 6:**
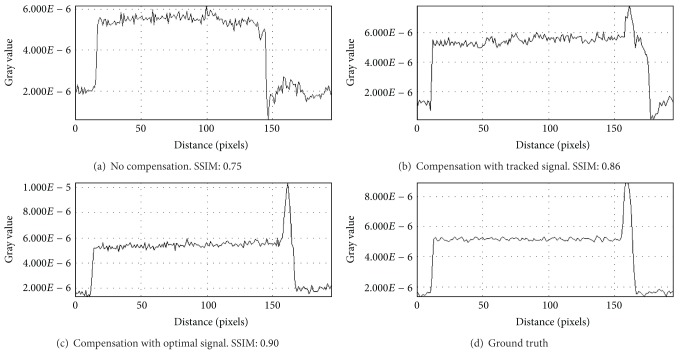
Line profiles of *xy*-slice 70 of compensated and uncompensated volumes (cf.  Figure 3(a)). Line profiles were taken at the position of the red lines in Figure 4.

**Figure 7:**
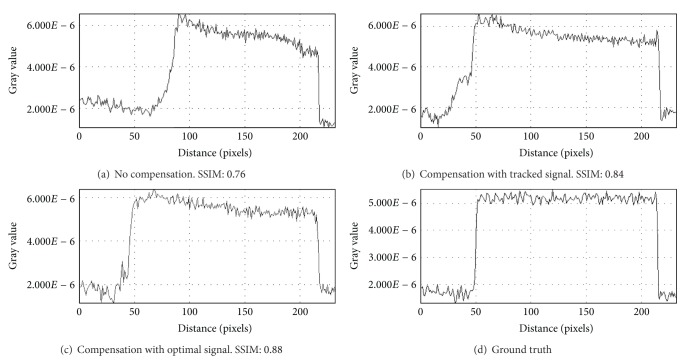
Line profiles of *xz*-slice 60 of compensated and uncompensated volumes (cf.  Figure 3(b)). Line profiles were taken at the position of the red lines in Figure 5.

For the evaluation of reconstruction quality we used the structural similarity index (SSIM) by Wang et al. [21]. SSIM was designed to improve on other methods such as mean squared error or peak signal-to-noise ratio. SSIM is a more accurate measure in terms of the perception of the human eye. SSIM measures the similarity of two images based on change in structural information, while traditional approaches are based on perceived errors. Two images are compared and a value between −1.0 and 1.0 is returned, where a value of 1.0 can only be reached when comparing identical images. In order to reduce the influence of the static background on the quality evaluation, the reconstructed volume was cropped to the bounding box that contains the heart. In total, we evaluated the quality of three different reconstructions: (i) a compensated reconstruction using the proposed tracking methods, (ii) a compensated reconstruction with the correct 2D projections of the diaphragm top (simulating an optimal diaphragm tracking), and (iii) an uncompensated reconstruction. For motion compensation we directly used the computed respiration signal, as the simulated motion in the XCAT phantom is constant for any given voxel.  Figure 3 shows the evaluation results for *xy*- and *xz*-slices. Both compensated reconstructions show highly improved image quality. As expected, the diaphragm tracking approach produces images slightly below the quality of the optimal reconstruction. However, it shows considerable improvement when compared to the uncompensated reconstruction. The uncompensated reconstruction seems to be superior in the first and last slices. The heart is only of small size in these slices. Therefore, the static background has a larger influence on the evaluation. Compensation blurs the static background which leads to a reduced SSIM, whereas it is perfectly reconstructed without motion compensation. Figures 4 and 5 show the results for two example slices. The red lines indicate the positions of the line profiles that are presented in Figures 6 and 7.

### 3.2. artiCHEST Lung Phantom

**Figure 8:**
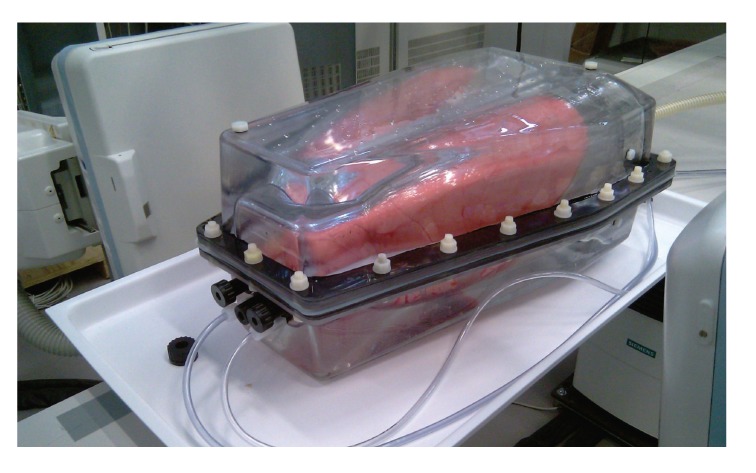
Illustration of the artiCHEST lung phantom. The box contains a porcine lung mounted on an artificial diaphragm and is filled with water.

**Figure 9:**
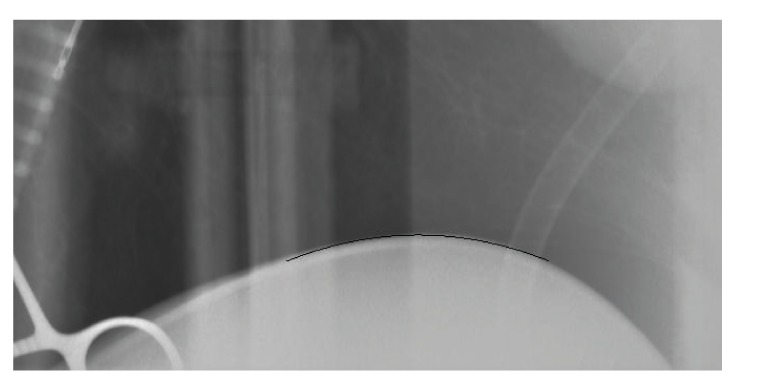
Zoomed in view of the diaphragm and the tracked function in a projection image.

 The second part of our evaluation will be focused on a pulmonary application. For this purpose we used the artiCHEST phantom (PROdesign GmbH, Germany). This phantom consists of a box with an artificial diaphragm. Inside the box a porcine lung is mounted that can be inflated and deflated by a computer controlled pump. In this manner, we can simulate previously configured respiration pattern. In our experiments, we set the respiration pattern to be sinusoidal with a maximum amplitude of about 1 cm. The borders of the box are filled with water.  Figure 8 shows the test setup. Since the phantom consists only of one centric diaphragm, we had to simplify the diaphragm tracking approach. We only used the standard approach, without any additional motion constraints since the diaphragm remains relatively static in the center of the projections. While previously in the XCAT phantom only a constant motion was simulated, the motion simulated in this lung phantom is closer to reality. The motion is approximately linear; less motion is observed at the top of the lungs compared to the region close to the diaphragm. As no ground truth data was available to evaluate the tracking performance, tracking quality could only be inspected visually. Tracking performance appeared as robust as in the case of the simulated XCAT phantom.  Figure 9 shows a cutout of an exemplary frame with the tracked parabola.

In the previous XCAT phantom test, the correlation of diaphragm and heart motion was already known. As previously noted, the relation of diaphragm and lung motion is typically linear. In order to approximate the linear scaling factor of our test lung, we acquired 11 static C-arm CT scans uniformly distributed along the simulated respiratory curve. Subsequently, we manually measured the position of the diaphragm top in each of these volumes to determine an optimal scaling for the *z*-coordinate correction; that is, we assume correct results in the triangulation in the following.

In order to approximate respiratory motion of the lung, we tested a simple linear motion model. While this model is obviously very simple, it already provides visible improvement to image quality. For clinical cases a simple model like this might only work in case of abdominal breathing. Compensation of thoracic breathing motion without prior knowledge is still an unsolved field of research. In this model, we estimated the slope of the linear function as

(4) $$\begin{array}{c} m=\frac{r_{i}}{\left(z_{\max}-z_{\text{dia}}\right)}, \end{array}$$

where *r*_*i*_ is the scaled diaphragm motion, *z*_dia_ is the *z*-coordinate of the diaphragm top in the current projection we are backprojecting, and *z*_max⁡_ is the *z*-coordinate of a manually chosen point at which we assume the motion to be zero. In our case we used the topmost point of the lung.

**Figure 10:**
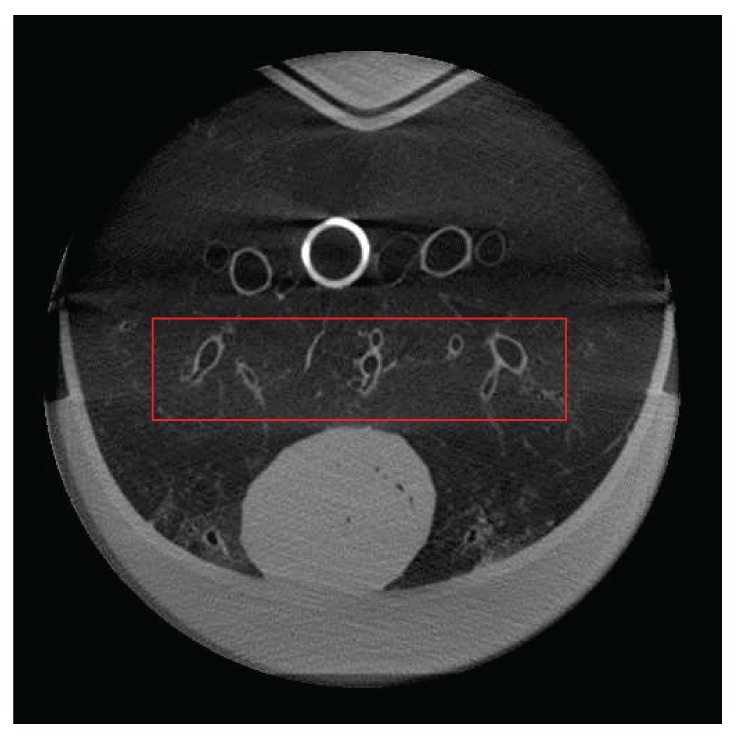
A slice of a static reconstruction of the artiCHEST phantom. At the bottom there is a porcine heart; the white circle is a plastic tube representing the spine. The red bounding box shows the region of interest for further evaluation.

Figure 10 shows a slice of a static reconstruction of the lung phantom without any motion present. In the lower part of the image the porcine heart is located. The heart has to be included in the phantom, as it provides stability. Unfortunately, this also means that the heart and its surrounding vessels and airways remain very static, due to the heart's mass and its location at the phantom border. The bright circular object in the center is a plastic tube that was included to simulate the spine and the metal artifacts to the left of the tube are caused by a pair of scissors that was included to mimic interventional constraints. Therefore, a meaningful evaluation can only be done in the center of the slice, which is depicted by the red bounding box in the image. 

**Figure 11:**
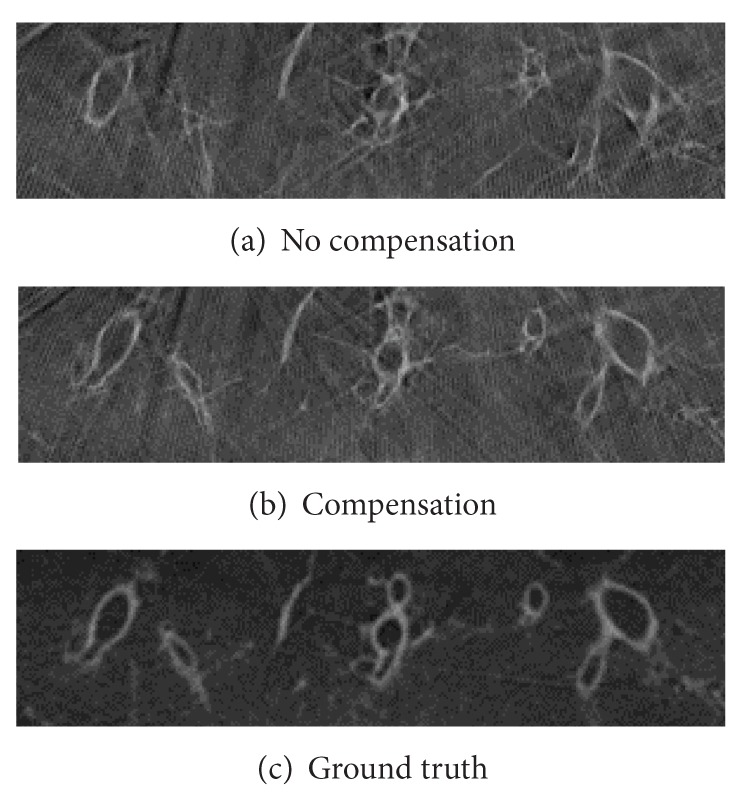
Detailed view of compensated and not compensated slices of a 20s scan with 2 respiration cycles of only 50% respiratory amplitude.

**Figure 12:**
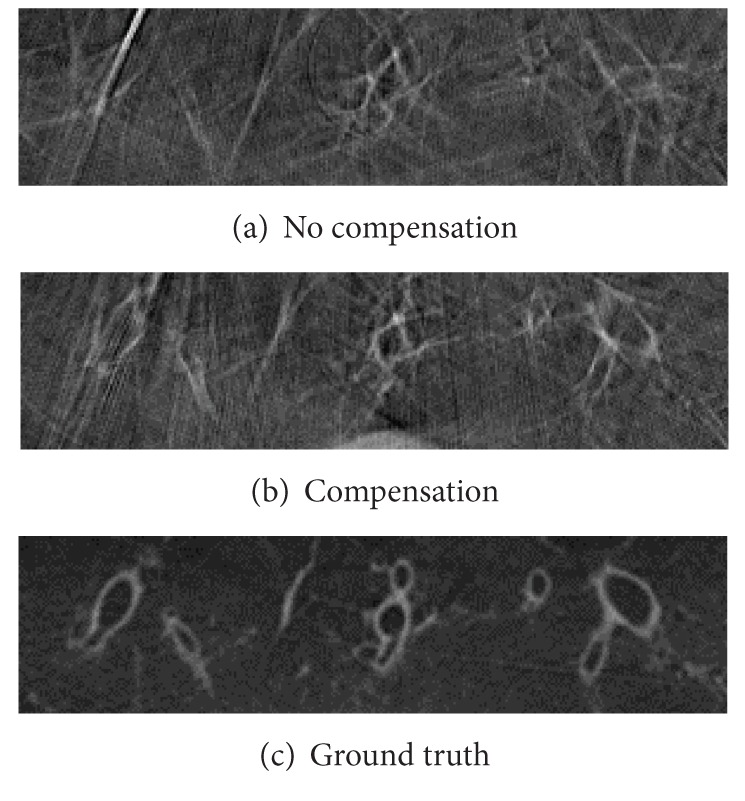
Detailed view of compensated and not compensated slices of a 20s scan with 2 full respiration cycles.

The motion model was tested on two 20s C-arm CT scans. During the first scan approximately two full respiration cycles were simulated with a reduced amplitude of the simulated respiratory motion of 50%. For the second scan, the amplitude of the simulated respiratory motion was increased to 100%.  Figure 11 depicts the red region of interest that was introduced in Figure 10 for this first case. For comparison, an uncompensated reconstruction and a static reconstruction of the phantom are shown. The compensated reconstruction shows improved image quality compared to the uncompensated reconstruction. The airways are severely distorted in the uncompensated volume, while after motion compensation the contours of the airways are clearly visible and at the correct positions.  Figure 12 shows the same slice for the second case with the full respiratory amplitude. While in the uncompensated image the airways are not visible at all, the compensation is able to restore the image partly. Compared to the static reconstruction, image quality is slightly degraded; however, this is still a notable improvement compared to the uncompensated reconstruction.

## 4. Conclusions

 We have shown for two exemplary applications that our respiratory motion compensated reconstruction already shows promising image quality improvement with the current simple motion model assumptions. As shown in the simulated phantom data, the diaphragm tracking is already able to improve image quality considerably from an SSIM of about 0.7 to an SSIM of 0.85. Compensation with an ideal triangulation result could improve image quality further to 0.9. Thus, future work must focus on the improvement of the diaphragm triangulation. At present, we only use the topmost point of the tracked contour to reconstruct the diaphragm top. However, as the topmost vertex point is not necessarily projected onto the diaphragm contour in the projection, the triangulation with the topmost point of the tracked contour may be erroneous. We expect improved reconstruction results if not only the topmost point but the complete diaphragm surface is reconstructed. In this manner, perspective projection problems are modelled correctly and more accurate results will be obtained.

Another major issue of our current approach is the linear motion model. It yielded already an improved reconstruction. However, compared to ground truth data, image quality was still reduced. With this respect, we regard our approach as an initial guess that can be computed in a straight forward manner, as we only require the diaphragm to be tracked in the projection data. Application of the motion correction is straightforward and yields an improved reconstruction. In this respect, our method is comparable to ECG-gating in motion compensation of the heart. The method works rather well, if the model assumptions are met (in ECG gating, this is a regular heart beat). As soon as the observed data violates these assumptions, image quality is degraded. As shown by various authors, this does not mean that ECG gating is not of any clinical applicability, although most cardiac patients do not have a regular heart beat. Instead, the method is applied in many advanced cardiac motion compensation scenarios as an initial guess that is then used in an additional method to improve image quality further. Thus, we believe that our method will be applicable as well as an initial estimate for such advanced methods in respiratory motion compensation.

In addition, our method is not limited to only cardiac or pulmonary C-arm CT. The approach can also be applied in other applications where respiratory motion is present, for example, liver C-arm CT, as long as the diaphragm is visible and a sufficient motion model can be given.

## Figures and Tables

**Algorithm 1:**
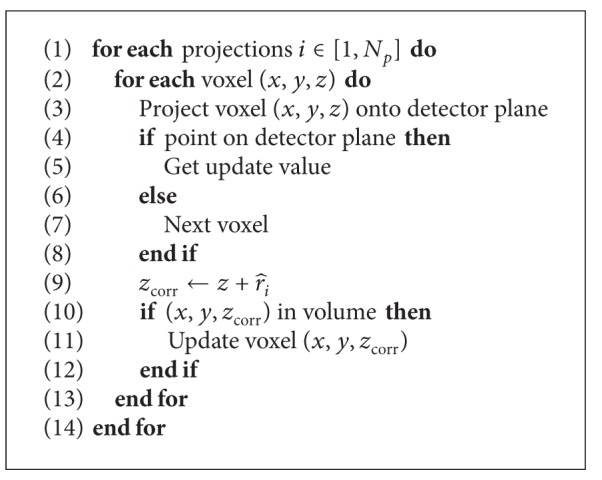
Motion compensated reconstruction. Respiratory motion is compensated in lines (9)–(11).

**Table 1:** Triangulation errors (in mm) based on projections of the real diaphragm top. Angular offset of the triangulated image pair in brackets.

	Mean 3D	Std. Dev. 3D	Mean *Z*	Std. Dev. *Z*
Rect. Iter. (90°)	0.20	0.06	0.10	0.06
Rect. Iter. (30°)	0.32	0.15	0.10	0.06
Rect. Iter. (10°)	0.89	0.60	0.11	0.08
Iterative (90°)	2.22	0.97	2.22	0.96

## References

[1] Jahnke C, Paetsch I, Achenbach S (2006). Coronary MR imaging: breath-hold capability and patterns, coronary artery rest periods, and *β*-blocker use. *Radiology*.

[2] Blondel C, Malandain G, Vaillant R, Ayache N (2006). Reconstruction of coronary arteries from a single rotational X-ray projection sequence. *IEEE Transactions on Medical Imaging*.

[3] Prümmer M, Hornegger J, Lauritsch G, Wigström L, Girard-Hughes E, Fahrig R (2009). Cardiac c-arm CT: a unified framework for motion estimation and dynamic CT. *IEEE Transactions on Medical Imaging*.

[4] Taguchi K, Segars WP, Fung GSK, Tsui BMW Toward time resolved 4D cardiac CT imaging with patient dose reduction; estimating the global heart motion.

[5] Schaller C, Penne J, Hornegger J (2008). Time-of-flight sensor for respiratory motion gating. *Medical Physics*.

[6] Wiesner S, Yaniv Z Respiratory signal generation for retrospective gating of cone-beam CT images.

[7] Marchant TE, Price GJ, Matuszewski BJ, Moore CJ (2011). Reduction of motion artefacts in on-board cone beam CT by warping of projection images. *British Journal of Radiology*.

[8] Wang Y, Riederer SJ, Ehman RL (1995). Respiratory motion of the heart: kinematics and the implications for the spatial resolution in coronary imaging. *Magnetic Resonance in Medicine*.

[9] Sonke J-J, Zijp L, Remeijer P, van Herk M (2005). Respiratory correlated cone beam CT. *Medical Physics*.

[10] Kavanagh A, Evans PM, Hansen VN, Webb S (2009). Obtaining breathing patterns from any sequential thoracic X-ray image set. *Physics in Medicine and Biology*.

[11] Chen M, Siochi RA (2010). Diaphragm motion quantification in megavoltage cone-beam CT projection images. *Medical Physics*.

[12] Vergalasova I, Cai J, Yin F-F (2012). A novel technique for markerless, self-sorted 4D-CBCT: feasibility study. *Medical Physics*.

[13] Bögel M, Maier A, Hofmann HG, Hornegger J, Fahrig R Diaphragm tracking in cardiac C-Arm projection data.

[14] Fischler MA, Bolles RC (1981). Random sample consensus: a paradigm for model fitting 350 with applications to image analysis and automated cartography. *Communications of the ACM*.

[15] Fusiello A, Trucco E, Verri A (2000). A compact algorithm for rectification of stereo pairs. *Machine Vision and Applications*.

[16] Hartley RI, Sturm P (1997). Triangulation. *Computer Vision and Image Understanding*.

[17] Schäfer D, Borgert J, Rasche V, Grass M (2006). Motion-compensated and gated cone beam filtered back-projection for 3-D rotational X-ray angiography. *IEEE Transactions on Medical Imaging*.

[18] Segars WP, Mahesh M, Beck TJ, Frey EC, Tsui BMW (2008). Realistic CT simulation using the 4D XCAT phantom. *Medical Physics*.

[19] Maier A, Hofmann HG, Schwemmer C, Hornegger J, Keil A, Fahrig R (2012). Fast simulation of X-ray projections of spline-based surfaces using an append buffer. *Physics in Medicine & Biology*.

[20] Bögel M, Maier A, Hofmann HG, Hornegger J, Fahrig R Diaphragm tracking for respiratory motion compensated cardiac C-Arm CT.

[21] Wang Z, Bovik AC, Sheikh HR, Simoncelli EP (2004). Image quality assessment: from error visibility to structural similarity. *IEEE Transactions on Image Processing*.

